# Gender and Racial Representation Trends Among Internal Medicine Department Chairs from 2010–2020

**DOI:** 10.1007/s11606-022-07783-z

**Published:** 2022-10-28

**Authors:** Anita Samuel, Ronald M. Cervero, Steven J. Durning

**Affiliations:** Center for Health Professions Education, School of Medicine, Uniformed Services University, Bethesda, MD USA

**Keywords:** academic medicine, leadership, disparity, equity

## Abstract

**Background:**

Quality medical education, reduction in health disparities, and healthcare research that includes all members of society are enhanced by diversity in departments of internal medicine (IM). Research on increasing diversity within the academic medicine student body or faculty notes the important role of leadership. Yet, there is a scarcity in research into diversity in leadership.

**Objective:**

The purpose of this study is to go beyond aggregate numbers and answer the question: What is the level of parity representation, by gender and race, at department chair positions in academic IM departments?

**Design:**

A cross-sectional analysis of race/ethnicity and gender in IM medical school departments from 2010 to 2020 was conducted using data from the American Association of Medical College’s (AAMC) Faculty Roster. The proportion of IM department chairs to IM faculty by race/ethnicity for each year (2010–2020) was used to calculate the Leadership Parity Index (LPI) in this study. LPI by gender and by gender and race/ethnicity were also calculated for each year.

**Results:**

In aggregate numbers, Black or African American and Hispanic, Latino, or of Spanish Origin faculty remain under-represented in academic IM each making up, on average, approximately 4% of the total IM faculty. The LPI calculations revealed that faculty who identified as White were consistently over-represented as department chairs while Asian faculty were consistently under-represented in leadership and ranked lowest in leadership parity among the ethnic groups studied. The leadership parity index also showed that women faculty across all races were under-represented.

**Conclusion:**

Women and Asian faculty encounter a ceiling effect that may be at play in IM departments. While significant progress still needs to be made in the representation of under-represented minorities, the findings of this study show that aggregate data does not provide a true picture of equity and parity in Internal Medicine faculties.

**Supplementary Information:**

The online version contains supplementary material available at 10.1007/s11606-022-07783-z.

## INTRODUCTION

Quality medical education, reduction in health disparities, and healthcare research that includes all members of society are enhanced by diversity in departments of internal medicine (IM).^1,2,3,4^ As the largest subspecialty in medicine, IM trains the largest number of medical students and postgraduate trainees and produces a majority of physicians.^5,6^ Internal medicine (IM), therefore, has a key role in healthcare.

However, racial and gender disparities in academic rank and promotion in IM departments persist.^7^ While women are nearly equal to men in representation at instructor and assistant professor levels, their representation drops dramatically at higher faculty ranks.^7,8^ A deeper analysis of IM faculty data reveals more nuanced trends. When parsed out by gender, the percentage of under-represented in medicine (URM) female faculty remains consistently above that of URM male faculty.^9^ Such variations in the data lead to Ibrahim’s call for a more granular analysis of the data to highlight areas of disparity that need attention in IM.^6^

One area that needs further study is diversity within IM leadership especially at the level of department chairs. Diverse representation in leadership positions can help ensure that minority voices are included in policy decisions, thereby broadening the dialogue around diversity and inclusivity.^10^ This then has the potential to disrupt systemic inequities.^11^ Most research on increasing diversity within the academic medicine student body or faculty notes the important role of leadership.^1,10,12–14^ Yet, there is a lack of in-depth research into diversity in leadership and the need for such diversity.^10^ This lack of data negatively impacts structural change which requires a clear picture of the baseline and the desired state.

To address the gap in research and identify the baseline data on diversity in leadership at IM departments, we conducted an exploratory study on the racial/ethnic and gender parity representation at IM department chair positions. We analyzed the racial and gender parity between IM faculty and IM department chairs. This study sought to answer the question: What is the level of parity representation, by gender and race, at department chair positions in academic IM departments?

## CONCEPTUAL FRAMEWORK

In this study, we examined the data of all racial/ethnic and gender groups. We have used two distinct terms in this manuscript when referring to racial/ethnic and gender groups.

**Underrepresented in Medicine (URM)**: In 2004, the AAMC adopted the term “underrepresented in medicine” to refer to groups whose representation in medical schools falls below their representation in the general population of the USA.^15^ For example, Blacks and African Americans constitute approximately 13.4% of the general population of the USA.^16^ However, they only make up about 6.1% of medical school matriculants and they are, therefore, categorized as URM.^17^

**Marginalized**: Women and Asian faculty in medical education are no longer considered URM as their percentage representation in medical school faculty is comparable to (women) or more than (Asians) their percentage representation in the general US population. Therefore, we have chosen to use the term “marginalized groups” to refer to women and Asian faculty.

The over-representation of Asians in aggregate faculty numbers has moved them into the non-underrepresented category with Whites. The result of this categorization is that there is sparse research into Asians in academic medicine.^18^ While their numbers might preclude them from being classified as URM in academic medicine, Asians are viewed as “different” and experience discrimination and bias as other minority groups.^18^ Asian physicians experience ethnic and racially offensive remarks from patients and co-workers and have also experienced physical harm.^19^ Furthermore, representation of Asian and women faculty drops at higher academic levels and in positions of leadership.^20^ Asians and women encounter barriers in their career trajectory and are marginalized as they are excluded from positions of power.

In this study, we examined the data for URM and marginalized groups in medical education to provide a comprehensive overview of the current leadership landscape at IM departments in medical schools.

## METHODS

A cross-sectional analysis of race/ethnicity and gender of IM faculty and IM department chairs in IM medical school departments from 2010 to 2020 was conducted. We selected this time frame to provide a sufficient breadth of data to study current trends. Institutional Review Board clearance was not required as only publicly available de-identified data were used in this study. Findings are reported in accordance with the Strengthening the Reporting of Observational Studies in Epidemiology (STROBE) reporting guideline for cross-sectional studies.^21^

### Data Sets

The September 30, 2021, snapshot of the US Medical School Faculty (USMSF) data from the American Association of Medical College’s (AAMC) Faculty Roster was used as the data set. This study used 11 years of data to identify trends in minority representation at IM department chairs levels.

Data were extracted from two data sets: (1) the distribution of department chairs by department, sex, and race/ethnicity, and (2) US medical school faculty by gender, race/ethnicity, rank, and department. These data sets represent self-reported data compiled annually by AAMC from all medical schools in the USA. Data relevant to IM were extracted for analysis from these primary data sets. The AAMC data used the term “gender” which was classified as a binary value of *women* or *men*. We use this classification in this study. The race/ethnicity categories represented in the data sets were as follows: (1) American Indian or Alaska Native; (2) Asian; (3) Black or African American; (4) Hispanic, Latino, or of Spanish Origin; (5) Native Hawaiian or Other Pacific Islander; (6) White; (7) Other; (8) Multiple Race–Hispanic; (9) Multiple Race–Non-Hispanic; and (10) Unknown Race/Ethnicity.

The data for 2010–2020 comprised of 448,986 IM faculty (professors, associate professors, assistant professors, instructors, and others reported as faculty) and 1830 IM department chairs.

### Methodological Framework

Parity indices have been used to calculate the global gender gap, inform legislative initiatives on health equity, corporate leadership gaps, and rank equity in academic medicine.^22–26^ Parity studies in academic medicine have found that minority faculty are predominantly represented at lower academic ranks (instructor, assistant professor).^25,26^ Minority representation at the levels of associate and full professor or in leadership (department chairs and deans) are not in parity with their representation at the lower levels. Studies into parity move beyond aggregate numbers to provide more nuanced analysis of data.

This study used the Leadership Parity Index (LPI) adapted from the Executive Parity Index (EPI) as the unit of analysis. The EPI was developed in 2015 to assess parity in corporate workforce leadership representation.^24^ The EPI has also been adapted to calculate Rank Equity Indices (REI), examining the academic pipeline for faculty in medical schools.^25,26^ In this study, the Leadership Parity Index (LPI) is calculated as:

$$ Leadership\ Parity\ Index=\frac{A\  grou{p}^{\prime }s\  percentage\ representation\  as\  leaders}{\ {The\ group}^{\prime }\ s\  percentage\ representation\  as\  faculty} $$

Parity in the percentage of leaders and faculty is represented by an LPI of 1.00. Values below 1.00 indicate under-representation, and values over 1.00 indicate over-representation. Studies using parity calculations (gender parity, EPI, REI) comment on over-representation (parity index above 1) and under-representation (parity index below 1) with “1” being seen as the ideal.^22,25,26^

### Data Analysis

Descriptive statistics for the IM department chairs and faculty were calculated. The proportion of IM department chairs to IM faculty by race/ethnicity for each year (2010–2020) was used to calculate the LPI by race/ethnicity for each of the four race/ethnicity categories in this study. In addition, LPI by gender and gender and race/ethnicity were also calculated for each year.

## RESULTS

### Demographic Distribution

From 2010 to 2020 there were 448,986 IM faculty members and 1830 IM department chairs. White and male faculty were in the majority for both levels (faculty and department chairs). Black or African American faculty and Hispanic, Latino, or of Spanish Origin faculty remain under-represented in academic IM, each making up approximately 4% of the total IM faculty. American Indian or Alaskan Native faculty or Native Hawaiian or Other Pacific Islander faculty are also under-represented constituting only 0.1% of IM faculty. (See Table 1 for details.)

**Table 1:** Racial and Gender Distribution of IM Faculty and Department Chairs from 2010 to 2020

	Faculty number (%)	Department chairs number (%)
American Indian or Alaskan Native	483 (0.10%)	0 (0%)
Asian	102834 (22.9%)	142 (7.75%)
Black or African American	15818 (3.52%)	82 (4.48%)
Hispanic, Latino, or of Spanish Origin	14665 (3.26%)	100 (5.46%)
Native Hawaiian or Other Pacific Islander	524 (0.11%)	0 (0%)
White	278263 (67.61%)	1443 (78.85%)
Other	4013 (0.89%)	17 (0.93%)
Multiple Race–Hispanic	10995 (2.44%)	3 (0.16%)
Multiple Race–Non-Hispanic	9632 (2.14%)	37 (2.02%)
Unknown	11759 (2.61%)	6 0.32%)
Women	172852 (38%)	264 (14%)
Men	276134 (62%)	1566 (86%)

### Race/Ethnicity LPI

**Fig. 1:**
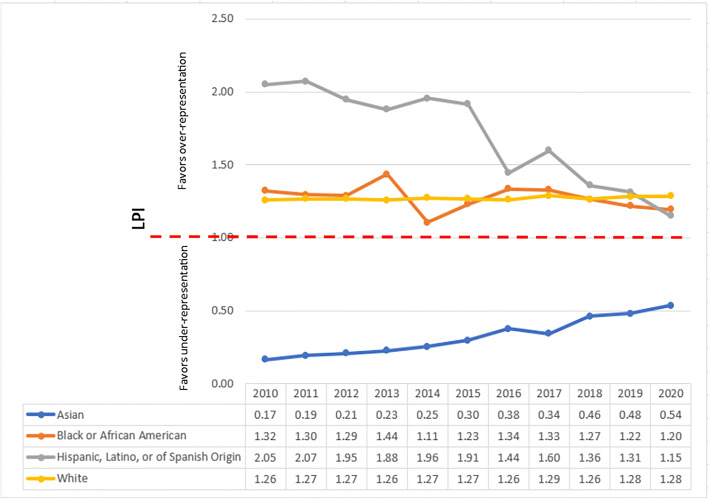
Leadership Parity Indices (LPIs) of IM department chairs by race/ethnicity.

From 2010 to 2020, White and Black or African American faculty have achieved leadership parity index of 1 as IM department chairs. While the leadership parity index for Hispanic, Latino, or of Spanish Origin faculty has been moving downwards since 2010, it still remains above 1. Asian faculty, however, have remained under-represented with LPI ranging from 0.17 in 2010 to 0.54 in 2020 (a 46% gap in achieving parity representation). (See Fig. 1.)

**Fig. 2:**
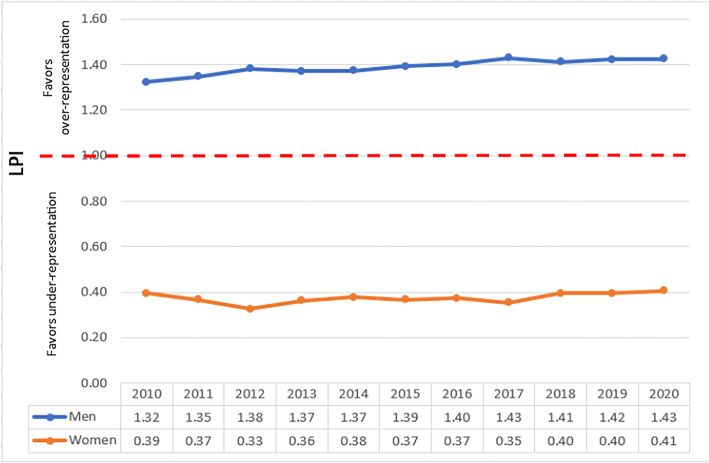
Leadership Parity Indices (LPIs) of IM department chairs by gender.

**Fig. 3:**
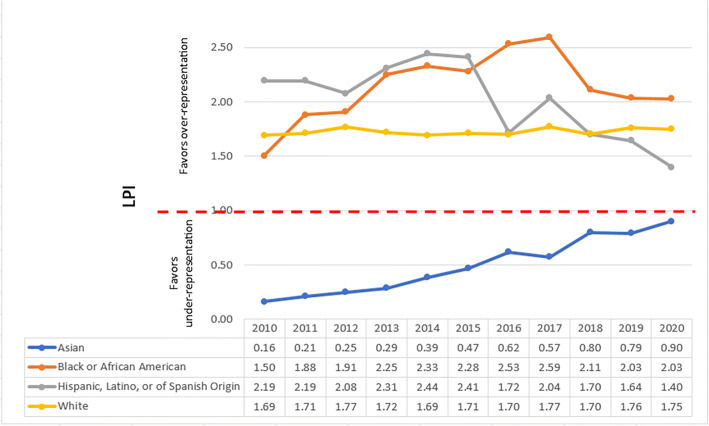
Leadership Parity Indices (LPIs) of male IM department chairs by race/ethnicity.

**Fig. 4:**
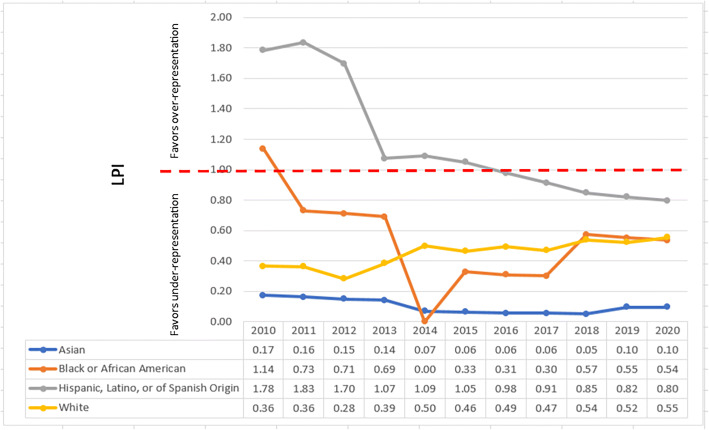
Leadership Parity Indices (LPIs) of female IM department chairs by race/ethnicity.

From 2010 to 2020, there have been no American Indian or Alaska Native or Native Hawaiian or Other Pacific Islander department chairs. Therefore, data in these two categories have been removed from Figs. 1, 2, 3, and 4. Data about Other, Multiple Race–Hispanic, Multiple Race–Non-Hispanic, and Unknown Race/Ethnicity have also been excluded to maintain clarity in the representation of ethnicities. Figures with all the groups are available in Supplemental materials 1.

### Gender LPI

In IM department chairs, from 2010 to 2020, men are consistently over-represented and women, consistently under-represented. LPI for IM women has stayed at or below an LPI of 0.4 while the LPI for men has moved between 1.32 in 2010 and 1.43 in 2020 (Fig. 2).

### Gender and Race LPI

Across the four ethnic groups of White, Asian, Black or African American and Hispanic, Latino, or of Spanish Origin, Asian men are the only group under-represented as IM department chairs with LPI ranging from 0.16 (2010) to 0.90 (2020). (See Fig. 3.)

While women faculty as a group are under-represented as IM department chairs (see Fig. 2), when studying the data for only women faculty, White and Asian women faculty have been under-represented across the 11 years. Hispanic, Latino, or of Spanish Origin women faculty representation as department chairs has seen more movement beginning with over-representation in 2010 and ending at under-representation in 2020. (See Fig. 4.)

## DISCUSSION

This study reveals patterns and potential gaps that exist in parity representation of racial/ethnic and gender groups in positions of IM department chairs at medical schools. When compared to the general population, the under-represented in medicine (URM) faculty remain under-represented both as faculty and department chairs in IM departments. The parity calculations in this study, however, compared percentage representation within IM department faculty and department chairs. These LPI calculations present a different picture.

In comparison to their representation within IM faculty, URM faculty in this study are at parity representation as IM department chairs. IM departments seem to be making conscious efforts to diversify representation at leadership positions. Hence, over the last 11 years, Hispanic, Latino, or of Spanish Origin and Black or African American faculty representation in leadership has been at or above parity with their numbers within the faculty. However, it should be noted that almost 16% of Black or African American faculty are at the three historically Black institutions. Therefore, the parity distribution is likely not equally distributed across all academic medicine institutions.

The representation of women and Asian faculty in IM leadership is different. While women are almost equal to men in aggregate numbers in IM faculty, they fall below parity representation at department chair positions. Over the past 11 years, the LPI for women faculty has not moved beyond 0.4, indicating a 60% gap in achieving parity in department chair representation.

In 2020, Asian faculty made up about 25% of the IM faculty but only 8% of IM department chairs. Over the 11 years studied, Asian faculty have consistently fallen below parity representation in leadership. When parsed out by gender, both Asian men and Asian women faculty are below parity compared to their representation within the IM faculty. But the representation of Asian men faculty as IM department chairs has been moving towards parity from 0.16 in 2010 to 0.9 in 2020. However, Asian women faculty have never exceeded a parity of index of 0.17 which was in 2010.

These findings show that discrimination can occur in different ways and representation in aggregate numbers does not tell the whole story.^25,26^ As Wesson et al. note, “Discrimination is often subtle but pervasive. It often appears as unrecognized assumptions and attitudes that work systematically against minorities and women.”^14^

The under-representation of Asian and women faculty in IM leadership is concerning given the ramifications. Limiting access to leadership can be a manifestation of discrimination as certain voices are excluded. The practical implications of this are that not all perspectives are equally considered in decision making processes and the process itself begins to lack credibility.^10^ This also engenders non-inclusive learning environments.^27^

Diversity in faculty and leadership is a visible demonstration of an institution’s commitment to diversity and a testament to what is possible.^8^ The lack of parity in representation for women and Asian faculty in IM department chair positions conveys a concerning message that while diversity is encouraged, there may be a ceiling on the achievement of certain groups. There is an implicit institutional message that it is not possible for members of these groups to move into IM department chair positions and the gap between espoused values and actual practice is revealed.^10^

The terminology of “underrepresented in medicine” has enabled institutions to develop focused programs to address issues that are specific to this population. Despite these initiatives, there have been no American Indian or Alaska Native and Native Hawaiian or Other Pacific Islander department chairs from 2010 to 2020. The diversity initiatives to draw more URM into academic medicine need to be more robust.

The unintended consequence of the term URM has been that Asian faculty are now placed in the same category as the majority whites. Asians are seen as the “model minority”^28^ who are white adjacent and “WASPs with brown skin.” ^29^. Yet, their experiences of discrimination show that they do not share the privilege of the majority.^19^ Asian faculty find themselves de-minoritized and occupying a liminal space neither URM nor white. The findings from this study highlight the disparities that can creep in when the primary focus is placed on representation in terms of aggregate numbers. Representation in aggregate numbers can lead to a sense of complacency wherein more subtle forms of discrimination are overlooked.

This study also calls into question the use of broad classifications such as URM or Asian. Asian is an umbrella term representing diverse communities from the affluent Indians and Chinese to the poorest populations in the USA (Hmong, Vietnamese, and Cambodian).^30^ Asian groups such as the Hmong are far from being over-represented in medicine. Yet, their situation is completely overlooked. Broad classifications such as URM or non-URM can hide discrimination that is experienced and manifested in ways that are specific to each sub-group.

Calculations such as the leadership parity index (LPI) used in this study can help provide a nuanced picture of the baseline of the leadership landscape in IM departments. The LPI could help identify gaps in the IM leadership pipeline by revealing groups that are being marginalized within departments and guide interventions to increase the diversity of department chairs.^31^

## LIMITATIONS OF THE STUDY

This study is based on datasets obtained from the AAMC faculty roster and shares the limitations of the dataset. The data set only provided IM leadership in the role of department chair. Therefore, other leadership roles such as associate deans, program/course directors, etc. could not be explored. But the findings of this study begin a conversation about the lack of parity representation at the level of department chairs.

Furthermore, the datasets only identified “men” and “women” and did not allow for a deeper analysis of other gender identities. This study highlighted the consequences of using broad categorizations and the gender categorization in this dataset could be obscuring other patterns that need to be studied.

We recognize that there might be variation in gender and racial representation of department chairs based on regional location. However, the dataset we used provided only aggregate information and did not parse out the data based on geographical regions. Since this is an initial exploratory study into the trends in leadership representation, we decided to use this data. Future studies could replicate this study across different regions to identify other trends.

The lack of leadership representation could be due to various reasons including the fact that not all faculty might want or accept leadership positions. Further research is needed to examine why Asians and women are not in leadership positions.

This study used the parity index calculation which is fairly new in the medical education literature. Therefore, questions such as acceptable levels of variation from a parity of 1 have yet to be explored.

## CONCLUSION

Studying representation in IM leadership through a parity lens provides another perspective on diversity in medical education. Women and Asians encounter various challenges in their progression to leadership positions which have been termed the “glass ceiling” and “bamboo ceiling” respectively. This study shows that a ceiling effect may also be at play in IM departments.

The Hispanic, Latino, or of Spanish Origin and Black or African American URM faculty groups have attained parity representation in department chairs at IM departments. While IM departments are showing movement in a positive direction, this study shows that we cannot become complacent in our efforts at diversity. Issues of discrimination play out in different ways, and it is important for us to remain vigilant and work at uncovering hidden biases.

## Supplementary Information

ESM 1 (PNG 135 kb)

ESM 2 (PNG 128 kb)

ESM 3 (PNG 133 kb)

## Declarations

### Disclaimer

The opinions and assertions expressed herein are those of the author(s) and do not reflect the official policy or position of the Uniformed Services University of the Health Sciences or the Department of Defense.
