# Supplementary figures and images for: Gender and Racial Representation Trends Among Internal Medicine Department Chairs from 2010–2020
Source: J Gen Intern Med. 2022 Oct 28;38(4):898–904. doi: 10.1007/s11606-022-07783-z (PMC10039186; doi:10.1007/s11606-022-07783-z)

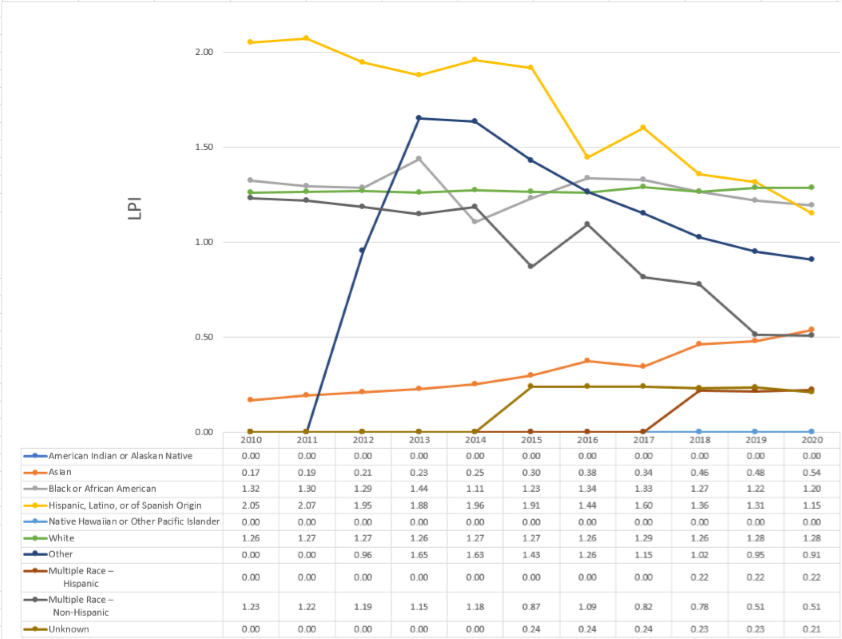

Supplement: Supplementary file 1 — (PNG 135 kb) [file 11606_2022_7783_MOESM1_ESM.png]

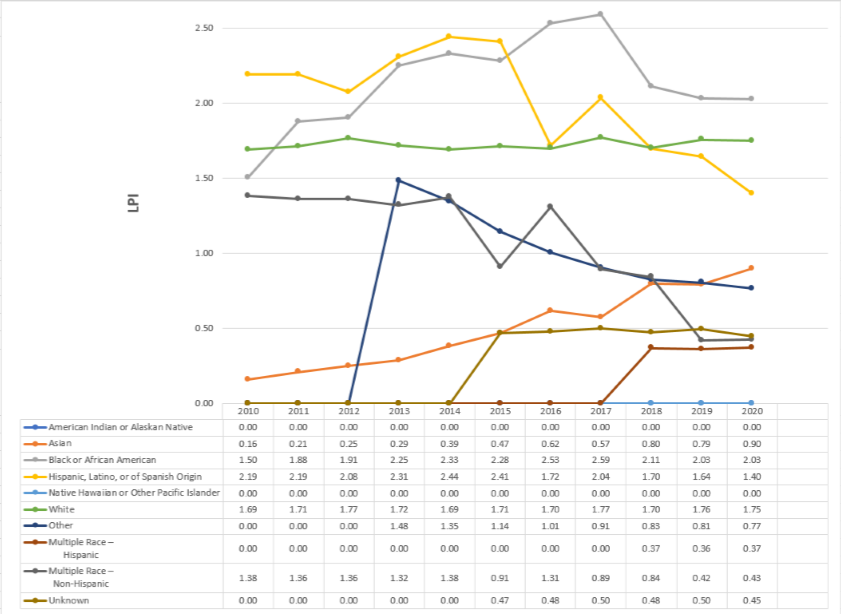

Supplement: Supplementary file 2 — (PNG 128 kb) [file 11606_2022_7783_MOESM2_ESM.png]

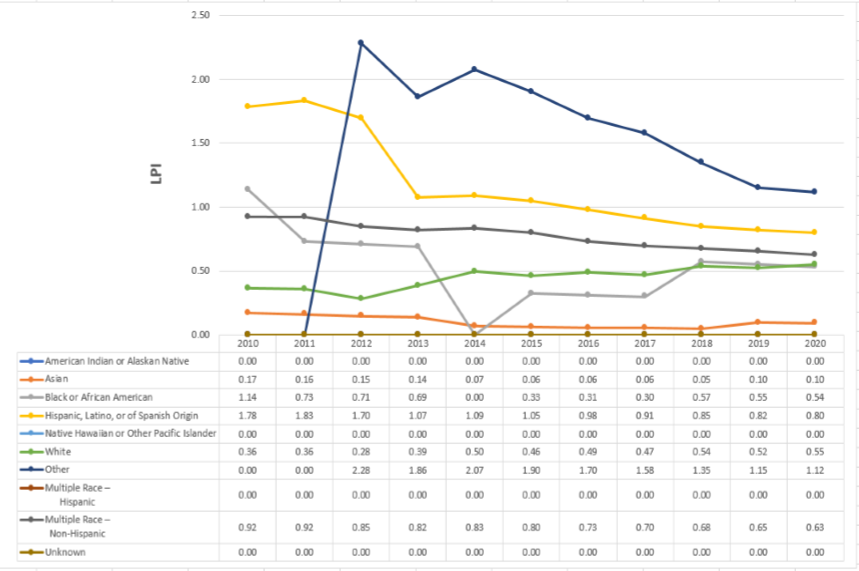

Supplement: Supplementary file 3 — (PNG 133 kb) [file 11606_2022_7783_MOESM3_ESM.png]
